# Distinct pattern of mutations of conserved regions of TP53 in colorectal cancer patients in the Kashmir population: an emerging high-risk area

**DOI:** 10.3332/ecancer.2009.129

**Published:** 2009-01-09

**Authors:** S Rehman, AS Sameer, L Zahoor, S Abdullah, ZA Shah, D Afroze, I Hussain, SM Shaffi, N Syeed, MA Rizvi, MA Siddiqi

**Affiliations:** 1 Department of Immunology and Molecular Medicine, Sher-I-Kashmir Institute of Medical Sciences, Soura, Srinagar 190011, Kashmir, India; 2 Department of Clinical Biochemistry, Sher-I-Kashmir Institute of Medical Sciences, Soura, Srinagar 190011, Kashmir, India; 3 Department of Biosciences, Jamia Millia Islamia, New Delhi, India

## Abstract

**Background:**

Colorectal cancer (CRC) is one of the leading causes of mortality and morbidity. The Kashmir valley, in Northern India, has been described as a high-risk area for colorectal cancer.

**Aim:**

The aim was to make a preliminary attempt to study mutations in exons 5–8 (the DNA binding domain) of the tumour suppressor gene ***TP53*** in 42 CRC patients from Kashmir.

**Materials and methods:**

The study population consisted of 42 patients diagnosed with colorectal cancer. Mutations in exons 5–8 of the ***TP53*** gene were detected by means of single-strand conformation polymorphism (SSCP). All samples that showed different band migration patterns in the SSCP were confirmed by sequencing.

**Results:**

The 28 mutations were found in the ***TP53*** gene in 19 patients, comprised 23 substitutions (17 transitions + six transversions), and five insertions. The 23 substitutions represent 18 missense mutations, leading to amino acid substitutions, two nonsense mutations, leading to stop codons, while the remaining three were silent mutations. The five insertions represented frameshifts. Two of 28 mutations (7.14%) have not been previously reported in colon cancer samples and were identified as novel ***TP53*** mutations. Comparison of the mutation profile with other ethnic populations and regions reflected both differences and similarities indicating co-exposure to a unique set of risk factors.

**Conclusions:**

Mutation of the ***TP53*** gene is one of the commonest genetic changes in the development of human colorectal cancer. The high frequency of ***TP53*** gene mutations implicates ***TP53*** as a predominant factor for colorectal cancer in the high-risk ethnic Kashmir population.

## Introduction

Colorectal cancer (CRC) is the third most common cause of cancer-related death in the western world. The annual incidence of CRC worldwide has been estimated to be at least half a million [1]. It is a commonly diagnosed cancer in both men and women. In 2008, an estimated 148,810 new cases will be diagnosed, and 49,960 deaths from colorectal cancer will occur [2]. The development of CRC is a multi-step process, which can arise due to the accumulation of mutations in various different oncogenes, tumour suppressor genes and/or from epigenetic changes in DNA [3]. Recent progress made in the field of molecular biology has shed light on the different alternative pathways involved in the colorectal carcinogenesis, and more importantly the crosstalk among these pathways [4,5]. Mutations in the ***TP53*** tumour suppressor gene are identified in approximately 35–45% of CRC [6,7,8,9], and it may be associated with worse prognosis and chemo/radio resistance [10,11].

***TP53*** mutations are the most frequently detected genetic alteration in human cancer [12,13]. The ***TP53*** gene is located on the short (p) arm of chromosome 17, and 17p deletions are found in 6–25% of colonic adenomas and in as many as 75% of colonic carcinomas [14,15]. The ***TP53*** gene encodes a protein, which maintains genomic integrity by inducing cell cycle arrest and apoptosis when DNA is damaged [16]. Mutations in ***TP53*** gene occur in almost half of all CRCs, proposed as a late event in the transition of an adenoma to carcinoma [17]. The mutations in ***TP53*** are thought to cause an increase in the half-life of the protein, and in one study [18] are associated with its’ over-expression in the nucleus. Also, most of the mutations in ***TP53*** gene occur in exons 5–8, in highly preserved regions, and in the three main structural domains of the ***TP53*** protein (L2, L3 and the loop-sheet-helix [19]. These mutations cause the synthesis of a stable protein that loses the ability to bind DNA and activation of target genes [20]. Eighty per cent of the alterations that occur in CRC are nonsense mutations: GC to TC transitions occurring in CpG dinucleotides [21].

The effect that the different alterations located in the different exons on the prognosis of patients with CRC has not been widely studied, although Borrensen-Dale *et al* showed that the mutations affecting the L3 loop were related with a worse prognosis [19]; nevertheless, other authors have not been able to confirm these conclusions [22]. Other studies suggest that the different mutations in ***TP53*** mat affect the sensitivity of the tumours to treatment.

Therefore, the aim of our study was to assess the contribution of ***TP53*** gene mutations in incidence and development of colorectal cancer in patients from the Kashmir valley, since such data from this region are not available in the literature. Furthermore, we compared the mutation pattern in ***TP53*** from the present study with the International Agency for Research on Cancer (IARC) ***TP53*** database, as well as the data reported from various regions to dissect out the differences and similarities in the mutation profile.

## Materials and methods

### Patient specimens

From 56 patients who were diagnosed with CRC by clinicians using sigmoidoscopy and colonoscopy, tissue specimens from 42 colorectal cancers were obtained with consent from patients who underwent curative surgical resection from January 2005 to 2007 at the Department of General Surgery, Sher-I-Kashmir Institute of Medical Sciences, Srinagar, India. An experienced pathologist selected a representative tumour block and surrounding normal mucosal tissue block for each case. The patient group included 13 women and 29 men with ages ranging from 55 to 82 years. Overall, 22 tumours were localized in the colon and 20 in the rectum. Clinical diagnosis was confirmed in all cases by histological examination. The study protocol was approved by the Research Ethics Committee of the Sher-I-Kashmir Institute of Medical Sciences.

### DNA isolation

Genomic DNA was extracted from single-cell suspension of blood and tissue samples of colorectal cancer patients by standard Proteinase-K digestion and phenol/chloroform extraction for examining mutations in ***TP53*** [23].

### PCR-SSCP analysis

Exons 5–8 of the ***TP53*** gene encoding the DNA binding domain were amplified using four specific oligonucleotide primers (Table 1). PCR was performed in a 25-μl total volume reaction mixture containing 50 ng of genomic DNA, 100 ng of each primer, 100 μM of each NTP, 1.5 mM MgCl_2_, 1X of Taq buffer and 0.1 unit of Taq DNA polymerase (Fermentas). PCR was performed using the following conditions: initial denaturation at 95°C for two minutes, 35 cycles of denaturation at 95°C, annealing at 52–62°C (see Table 1) and extension at 72°C for one minute each and final extension at 72°C for 5 minutes in Biorad cycler. Negative controls (DNA was replaced with water) were amplified by PCR and included in each experiment. The PCR products were separated on a 2% agarose gel and analysed under a UV illuminator.

The SSCP analysis of the amplicons of exons 5–8 was performed on 6% non-denaturing polyacrylamide gels (PAGE), utilizing either non-radioactive silver staining or radioactive procedures [24,25]. For non-radioactive SSCP analysis [26], PCR products in denaturing buffer (95% formamide, 10-mM NaOH, 0.05% xylene-cyanol FF and 0.05% bromophenol blue) in 1:1 ratio were heat denatured at 95°C for five minutes, immediately cooled on ice for 20 minutes and 6 μl of each was loaded onto a 6% PAGE and electrophoresed in 0.5X Tris-borate EDTA buffer at ±17°C at 4 W constant power for 18–22 hours. Gels were then silver stained. For radioactive SSCP analysis, radio-labelled PCR products (using α32-pCTP) were added to denaturing buffer (95% formamide, 20 mM EDTA, 0.05% xylene-cyanol FF and 0.05% bromophenolblue) in a 1:10 ratio and were heat denatured at 95°C for 5 minutes, 3 μl of which were loaded on 6% PAGE and electrophoresed at 4 W in 0.5X Tris-borate EDTA buffer at ±17°C for 18–22 hours. PCR-SSCP analysis was repeated twice for each sample to minimize the possibility of artefacts due to contamination or polymerase errors. Interpretation of the SSCP analysis was by the consensus of two investigators. The gel was then transferred onto 3 mm Whatmann paper, covered with Saran wrap and dried in a vacuum drier at 90°C for one hour. The Saran wrap was then replaced by x-ray film and kept at −70°C for 48 hours. The mobility shift in DNA bands were visualized by developing the x-ray film in a developer.

### Sequencing

Purified PCR products of the samples showing mobility shift on SSCP analysis and randomly chosen normal samples were used for direct DNA sequencing, using the automated ‘ABI prism 310’. To minimize the sequencing artefacts by PCR, products from at least two different PCRs were sequenced using forward and reverse primers with BigDye terminator cycle sequencing ready reaction mix (Applied Biosystems) based on fluorescence-labelled dideoxy nucleotides as chain terminators. Purified single-stranded extension products were then resolved on the ABI Prism 310 sequencer.

### Statistics

All statistical analysis was performed using SPSS software, version 12 (SPSS, Chicago, IL). The chi-square test was used to determine associations of the presence of ***TP53*** with various clinicopathological parameters and classical risk factors such as smoking habit. Statistical significance was considered when p≤0.05.

## Results

Forty-two CRC cases were analysed for ***TP53*** gene mutation (exon 5–8) by PCR-SSCP followed by sequencing. Analysis of these genetic alterations revealed 28 mutations in 19 of 42 cases analyzed (45%) (Table 2). Among these there were five frameshift mutations, 17 transitions and six transversions. Frameshift mutations were observed at codon 264 (exon 8), 304 (exon 8), 297(exon 8), 166 (exon 5) and 174 (exon 5), respectively. There were three silent mutations at codon 154 (exon 5), 222 (exon 6), 224 (exon 6) and two nonsense mutations at codon 196 (exon 6) and 192 (exon 6), respectively.

Analysis of the mutation spectrum revealed a number of salient and interesting features, which included high frequency of G:C to A:T substitutions. Among the 28 mutations, there were ten in exon 5, seven in exon 6, four in exon 7 and seven in exon 8. Mutations at codon 248 were detected in three cases, while mutations at codon 175 were detected in two cases (Table 4). Our results are consistent with previous reports on the prevalence of ***TP53*** mutations in CRC ranging from 42% to 67% in other parts of the world. Mutation effect data revealed a high percentage of missense mutations (23/28) (82.14%) compared to frameshift mutations (5/28) (17.85%). Also, mutation pattern data of ***TP53*** revealed a high percentage of G:C > A:T (at CpG + non-CpG sites) (12/28) (42.85%) and G:C > C:G (3/28) (10.71%), transition and transversion mutations, respectively. All the missense mutations occurred in heterozygous state except one at codon 221, which occurred in homozygous state.

A significant amount of mutations were found in exon 5 (35.7%), exon 6 (25%), exon 7 (14.28%) and exon 8 (25%), respectively. Twenty-eight per cent (8/28) of the ***TP53*** mutations were located at hotspot codons 175, 196, 245, 248 and 282, but no mutations were detected at the hotspot codon 273. A nonsense mutation at codon 196 never reported in colorectal cancer (Arg > Stop) was found in one tumour.

Taking into account classical risk factors and clinicopathological parameters on the presence of ***TP53*** mutations, there was a statistically significant increase (p=0.01; OR= 6.17; 95% CI=1.58–24.05) in the incidence of ***TP53*** mutations in smokers rather than non-smokers. A significantly higher frequency of ***TP53*** mutations was seen in rectal (75%) compared with colon (18.1%) cancers (p=0.01; OR=0.29; 95% CI=0.07–1.14). The study also showed a statistically significant increase (p=0.0001; OR=0.03; 95% CI=0.005–0.19) in the incidence of ***TP53*** mutations in Dukes’ Stage C and D when compared with A and B. The comparison did not show significant association with age, sex or dwelling (Table 3). In addition, by taking into account the specific functional and structural domains of ***TP53*** affected by the mutations, the cases were also classified as follows: four of 19 cases (21%) with mutations of the L3 loop; one of 19 cases (5.2%) with mutations of the LSH motif; and 14 of 19 cases (73.6%) with mutations outside L3 loop and LSH.

## Discussion

Mutations of ***TP53*** are found in approximately half of all CRC cases, with a higher frequency observed in distal colon and rectal tumours, and a lower frequency in proximal, mucinous and MSI+ tumours. Previous analyses on different types of tumours have shown that most of the ***TP53*** mutations (∼95%) affect exons 5–8, which code for residues 130–286, the most important region for the folding and, therefore, for the stabilization of the tertiary structure of the protein (core domain), and which contains the site-specific DNA-binding domain [28]. In our screen for mutations in exons 5–8 of ***TP53*** on genomic DNA from primary CRCs of 42 patients, we observed a mutation frequency of 45.2% (19 of 42), within the fairly wide range of values reported previously in CRC (23–61%) [29–34]. This variability may be the result of the different methods used to assess ***TP53*** mutations (SSCP, denaturing gradient gel electrophoresis, temperature gradient gel electrophoresis and direct sequencing), the type of tumour storage (fresh/frozen tissue and paraffin embedded blocks), an intrinsic tumoural heterogeneity, and, in addition, more specific features of the patient cohorts in the study, in particular, histopathological staging and grading of the tumour. In fact, patients at an advanced Dukes’ stage (C and D) and/or with poorly differentiated tumours (G3) generally present a higher rate of ***TP53*** mutations [34,35], which correlates with our study. In accordance with several other reports [29, 32, 34], 46.4% of all of the mutations observed in our series (13 of 28) were in four of the five highly conserved areas of the gene, which include two important regions for ***TP53*** binding to DNA. One of these contains the amino acids needed for DNA interaction, in particular those that are part of the L3 Zn-BD and of the LSH motif. In our own series, 14.2% (4 of 28) of the mutations occurred in L3 and 3.5% (1 of 28) in LSH, in accordance with the results reported by Borresen-Dale ***et al*** [36]. Our data confirm that arginines 248 and 273, which interact directly with DNA, are among the most frequently mutated residues (in our series: 10% and 0%, respectively). The second region, which when mutated results in loss of ***TP53*** DNA-binding capacity includes the amino acids located in L2, which are required for the folding and stabilization of the central domain [28]. Mutations in this area were observed more frequently in our own series (28.5%). Mutations at specific codons, such as codon 175, may be an indication of specific exposures to toxic agents or of genomic susceptibility. It has been observed, however, that mutations in codon 175 are more frequent in the colon than in the rectum, as previously described by Servomaa ***et al*** [37], correlating with our study. A peculiarity of the ***TP53*** mutations in Kashmiri CRC tumours was the lack of deletions (0% versus 6.57% in IARC) and a higher prevalence of insertions 17.85% versus 1.4% in IARC R12, release). In our study, G:C→A:T mutations were more frequent in the CpG sites (32.14%) then non-CpG sites (21.42%), nearly matching what was reported in the study from Delhi, India (unpublished data, personal communication). The high prevalence of G:C→A:T mutations was again an observation of interest in our study. However, to establish a correlation between the enhanced G→A transition (53.56%) and the presence of nitrosamines in foodstuffs consumed in Kashmir [38] needs further investigation. Alkyl nitrosamines can cause O^6^-alkyl guanine adducts and base misrepairing during replication, resulting in G→A (or C→T on the other strand of the DNA) transition [39] and are considered a major risk factor for the Chinese. G:C→C:G transition was comparatively high in Kashmiri samples (14.28%) versus 7.51% in IARC). The G:C→T:A transversion was observed to be confined to males who smoke. This type of mutation has been suggested to arise as a result of adducts formation at guanosine by metabolites of benzo(a)pyrene 7,8-diol-9,10-epoxide, a major tobacco carcinogen [40]. Interestingly, in our study, we found 35% of mutations occurred at the hotspot codons in rectal tumours as opposed to 17% in colon tumours, which is contrary to a collaborative study on CRC in which the frequency is 34% and 41%, respectively [41]. In addition, in our series, the frequency of transition and transversion mutations in rectal tumours was 59% and 23%, respectively, while in colon tumours was 67% and 17%, respectively, again contrary to a collaborative study on CRC in which there is no difference in the frequency of transition and transversion mutations between rectal and colon tumours [41]. The reasons for the comparably high frequency of transition mutations in colon tumours, and the high frequency of transversions in rectal tumours needs further investigation.

In summary, a significantly higher frequency of ***TP53*** mutations was seen in rectal (75%) compared with colon (18.1%) cancers (p=0.01; OR= 0.29; 95% CI=0.07–1.14). The reasons for this are unknown but may indicate a more important role for ***TP53*** mutation in the development of rectal compared with colon tumours. Also, significantly higher frequencies of ***TP53*** mutations were seen in smokers rather than non-smokers. Interestingly, 70.58% (12/17) of patients who smoke had mutations in ***TP53*** (p=0.01) compared to only 28% (7/25) of non-smokers. For each site, the more advanced Dukes’ C–D tumours contained higher frequencies of ***TP53*** mutations compared with Dukes’ A–B tumours (Table 3). The higher frequency of ***TP53*** mutations in Dukes’ C–D (77.27%) compared with Dukes’ A–B (10%) tumours suggests these mutations are associated with a more aggressive phenotype. In support of this, tumours with the poor prognostic features, that is vascular or lymphatic invasion, also showed significantly higher frequencies of ***TP53*** mutations in the overall CRC cohort [42].

To sum up, the high frequency of delirious nonsense, frameshift and missense mutations observed in the ***TP53*** gene in tumours of patients with clinicopathological features such as Dukes’ C–D and histopathogical grade II and III is a finding that assumes significance in view of the fact that these features reflect poor prognosis. The study, therefore, suggests ***TP53*** as a potential molecular marker and prognostic tool, at least in a subset of colorectal tumours. Nevertheless, these observations need further investigation on a larger cross section of the colorectal cancer patients with relevant controls.

## Figures and Tables

**Figure 1:**
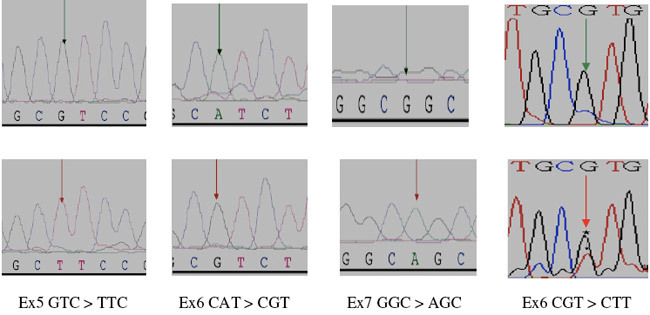
Partial electropherograms representing the normal (A) and mutant (B) (shown by asterisk and arrows), profiles in A of exon 5, in B of exon 6, in C of exon 7 and in D of exon 8 of *TP53*. 1A shows the transversion of G to T. 1B shows the transition of A to G. 1C shows the transition of G to A. 1D shows the transversion of G to T.

**Table 1:**
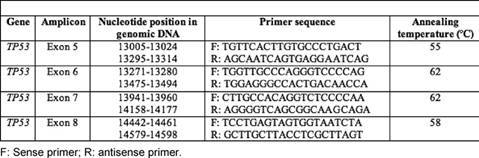
Primers used for screening different exons of TP53

**Table 2:**
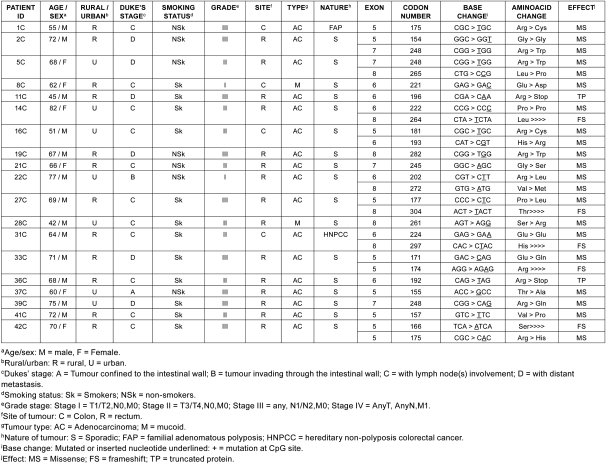
Details and nature of TP53 mutations in colorectal cancer patients from Kashmir valley

**Table 3:**
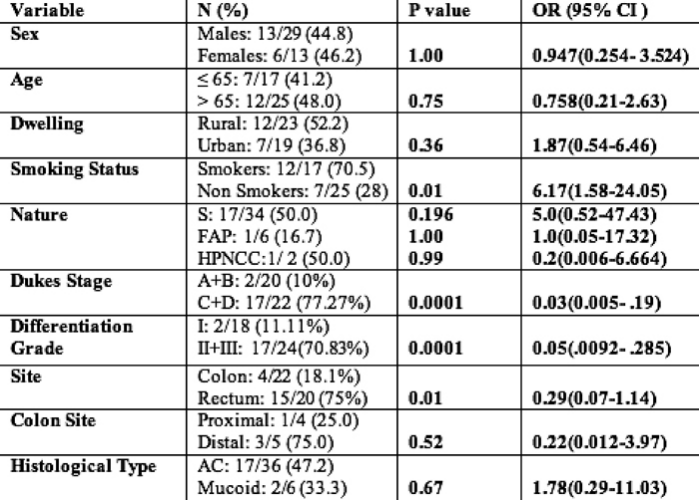
Association of *TP53* gene mutation with clinico-epidemiological features of 19 mutant colorectal cancer patients

**Table 4:**
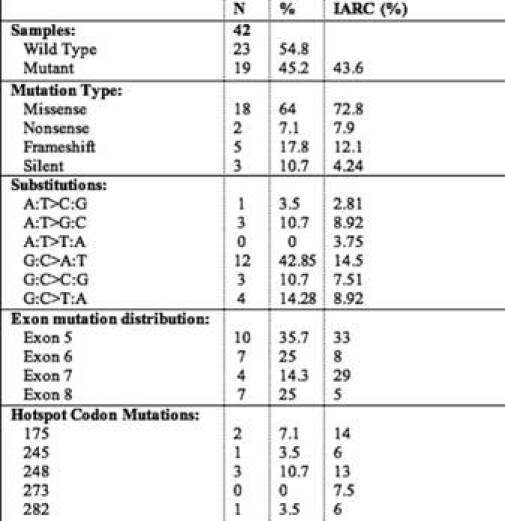
Mutation profile of the 19 colorectal carcinoma patients
